# A Plastic Torpedo-Shaped Firecracker That Caused Injury to the Parotid Gland: A Case Report

**DOI:** 10.7759/cureus.26297

**Published:** 2022-06-24

**Authors:** Yusuf Ç Kumbul, Vural Akın, Hasan Yasan, Bekir Büyükçelik, Yunus E Ekinci

**Affiliations:** 1 Department of Otorhinolaryngology and Head and Neck Surgery, Faculty of Medicine, Suleyman Demirel University, Isparta, TUR

**Keywords:** salivary gland surgery, trauma, ultrasonography, foreign bodies, parotid gland injury

## Abstract

Penetrating injuries affecting the parotid gland are relatively rare compared to injuries affecting other head and neck structures. Likewise, foreign bodies impacting the parotid gland are extremely rare. These foreign bodies can be secondary to penetrating injury or may reach the parotid gland by retrograde migration through Stensen's duct. Management of parotid gland foreign bodies is a challenging clinical situation for otolaryngologists due to the course of the facial nerve through the gland. In this article, a patient with penetrating parotid injury as a result of the explosion of a torpedo-shaped firecracker is presented. This object's injury to the parotid gland is presented for the first time in the literature. In addition, the importance of detailed physical examination was emphasized, as foreign bodies may embed under the skin in such injuries.

## Introduction

Penetrating injuries affecting the parotid gland are relatively rare. They usually occur as a result of work accidents and firearm injuries [1]. Foreign body (FB) retention in the gland secondary to penetrating parotid gland injuries is a much rarer clinical condition [2]. Parotid gland FBs can penetrate directly through the skin, or they can reach the gland retrogradely through the salivary gland ducts [3]. These injuries can lead to complications such as sialocele, cutaneous fistulas, and facial paralysis [1,3]. In addition, if the FB is not noticed, the inflammatory reaction triggered by this FB in the late period may mimic parotid tumors or sialadenitis [2-4].

In this case report, we present a female patient with a FB in the parotid gland that was noticed at a later time resulting from penetrating parotid gland injury for an unusual reason. Penetrating injuries of the parotid gland will be discussed along with this case in the light of the literature.

## Case presentation

**Figure 1:**
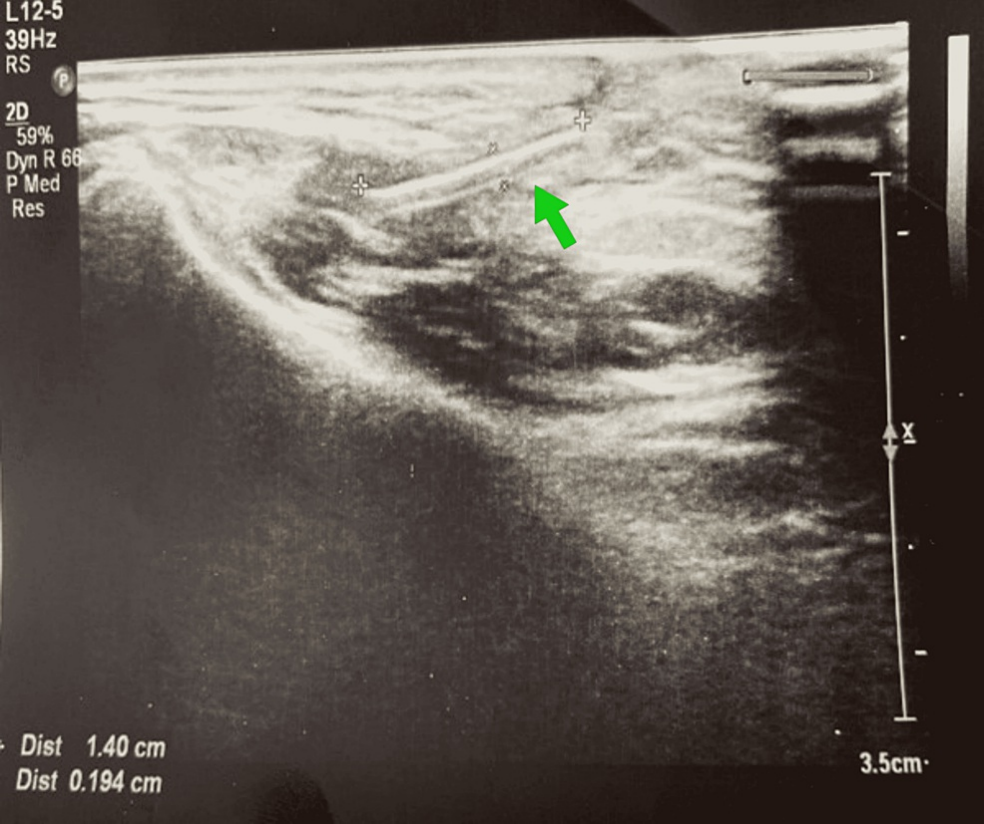
Ultrasonography image of foreign body Subcutaneous, associated with the parotid gland, approximately 14x2 mm in size, linear-shaped, hyperechoic image.

A 37-year-old female patient was admitted to our clinic with the complaint of stiffness under the skin in front of the right ear. While the patient was grazing sheep about 20 days previously, a wolf attack occurred and she used a torpedo-shaped firecracker to scare away the wolves. The patient stated that she was injured in front of her right ear during the explosion of the torpedo and attended the emergency department of a secondary-level state hospital. The patient, whose wound was dressed in the emergency department, was discharged with oral antibiotic therapy (amoxicillin/clavulanic acid, 2×1,000 mg, p.o.) and analgesic (paracetamol, 3×500 mg, p.o.) prescription. The patient used these drugs regularly for a week. About two weeks later, the patient, who developed swelling and pain in the wound area, was applied to the otorhinolaryngology department of the same hospital. On ultrasonography performed at the time of admission, the appearance of a 14x2 mm linear-shaped FB associated with the parotid gland was observed in the right preauricular region, and the patient was referred to us (Figure 1). 

**Figure 2:**
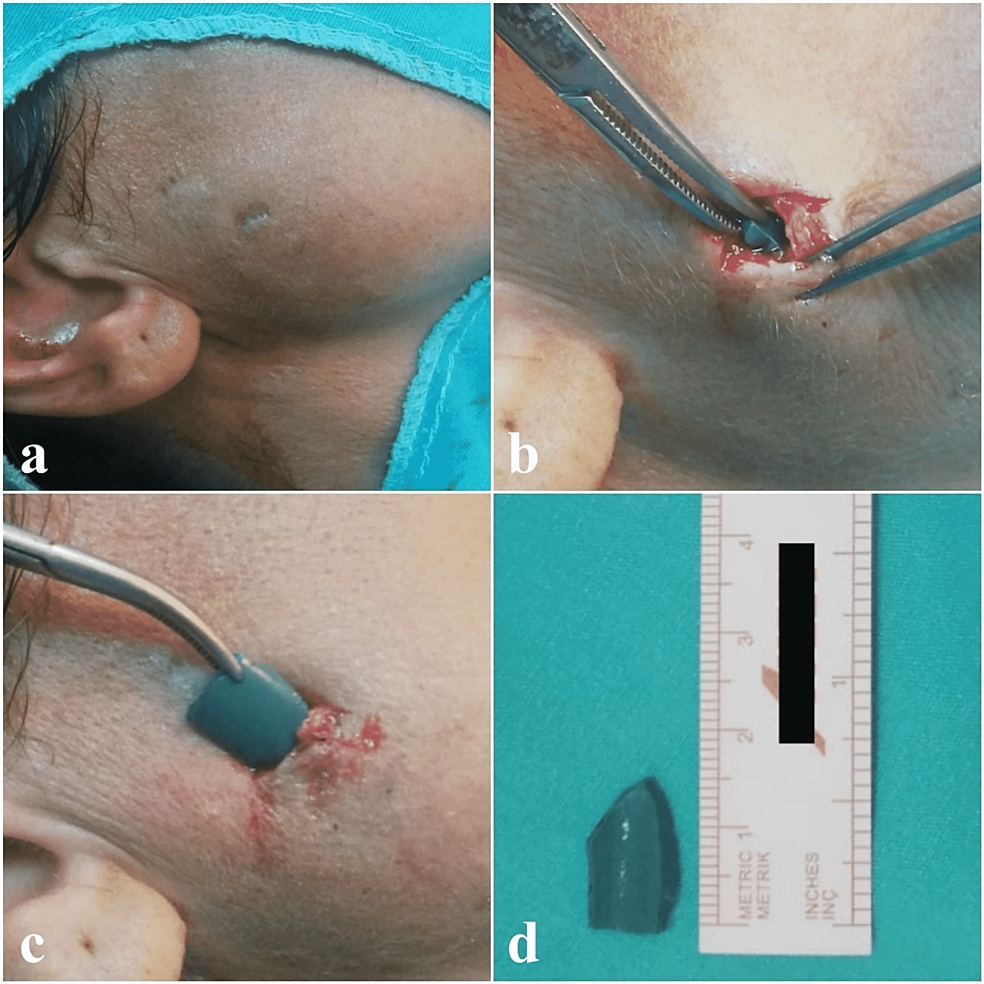
Images at the time of admission and during surgery The image of the scar at the time of admission (a). Intraoperative view of the foreign body (b, c); removed foreign body (d).

The patient had no history of surgery or disease in her past history. A skin scar of approximately 1 cm was observed on the right parotid gland (Figures 2a, 2b). Under the skin scar, an approximately 2 cm long, painful, well-circumscribed lesion was palpated. The facial nerve examination was normal. Intraoral examination revealed saliva flow from the Stensen’s duct. Complete blood count test, C-reactive protein, and biochemical tests were within normal limits. The decision was made to remove the FB under local anesthesia. With the incision made on the scar line, the skin and subcutaneous tissues were passed and the FB was reached. The dark green plastic FB was removed (Figures 2c, 2d).

**Figure 3:**
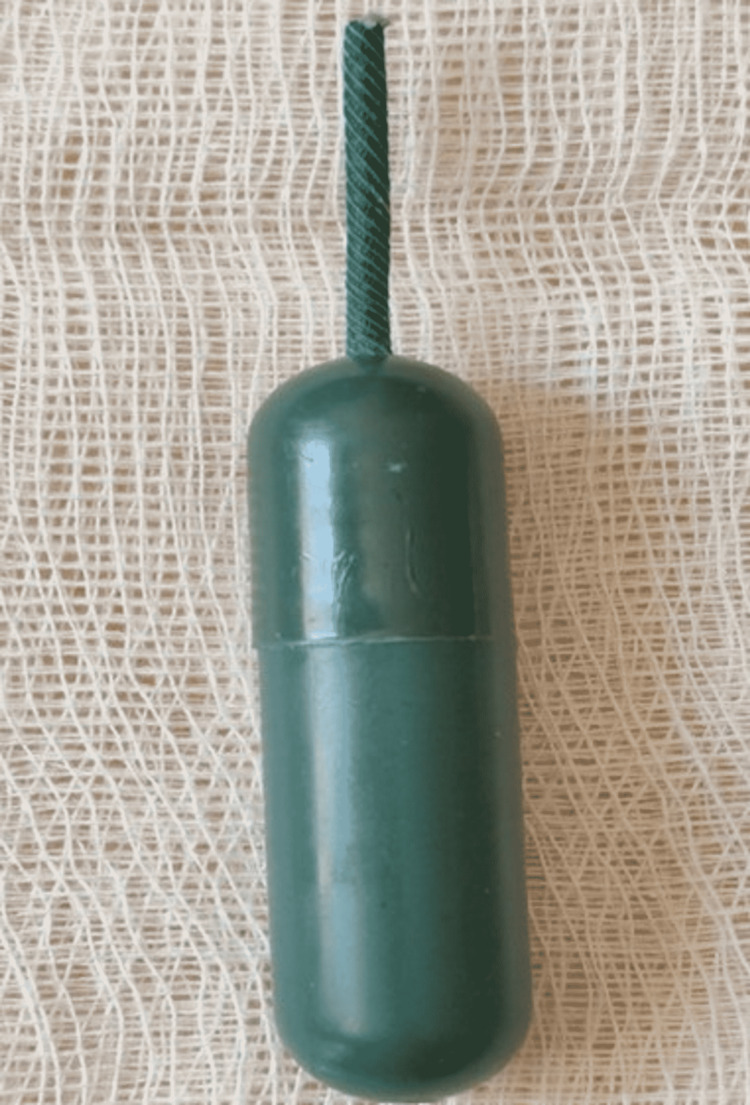
View of the torpedo-shaped firecracker that caused the injury

When the FB was shown to the patient, she stated that this object was part of the torpedo-shaped firecracker that she exploded in the wolf attack (Figure 3).

The cavity formed by the FB was irrigated with physiological saline and the incision line was sutured. The patient was discharged on the same day after the prescription of amoxicillin-clavulanate (1,000 mg tb; 2x1 p.o) and paracetamol (500 mg; 3x1 p.o). The patient had no complaints during the first week of check-up. The patient has been followed for three months and no complications have developed. Informed consent was obtained from the patient.

## Discussion

Parotid gland FBs are caused by direct penetration through the skin or retrograde migration through Stensen’s duct [3,5]. The first case was reported in 1958 [5]. In the cases in the literature, there are different materials such as metal, wood, glass, pencil tip, toothbrush bristle, fish bone, hair, grass, seed, and chicken feather among the FBs [3,5,6]. FBs reaching the parotid gland with retrograde migration are often localized in the duct system. These FBs can cause recurrent sialadenitis and sialolithiasis with the obstruction they create [3]. In untreated cases, fistulation to the skin may occur [7]. Xie et al. determined that the factor causing sialolithiasis was a fish bone in 13 of 333 sialolithiasis cases after they performed sialoendoscopy. All of these cases were located in the submandibular gland [6]. FB entry from the Wharton canal orifice is more common than Stensen’s duct [8]. FB obstruction of Stensen’s duct is extremely rare and usually occurs by the intraoral route. It is extraordinary for this to occur by the extraoral penetration route. Levine et al. reported that a ball-point pen tip, which was thought to have penetrated the skin three years previously, reached Stensen’s duct and caused suppurative parotitis. In this case, the FB was removed by intraoral incision [8].

If the penetrating injury is recent and there is a persistent infection, fistula, and trismus at the injury site, FB should be suspected [3]. In such cases, the most beneficial examination for the clinician is a detailed radiological examination. We think that computed tomography among radiological examinations is the most valuable as it can distinguish pathologies in the differential diagnosis, as well as contribute to the planning of the surgical procedure. Conventional radiographs are limited in use because some FBs are not radiopaque. Ultrasonography can be preferred in appropriate cases [5]. However, the experience of the radiologist performing ultrasonography may affect the result. FBs with soft consistency can be confused with other parotid lesions [7]. In the hospital where our case was first attended, the focus was on skin injury and the FB lodged under the skin went unnoticed. As the patient's complaints increased, FB was suspected and she was referred to us as a result of ultrasonography. Ultrasonography revealed that the FB was associated with the superficial lobe of the parotid gland. Therefore, no additional radiological examination was needed.

Parotid gland injury can often be overlooked or underestimated in patients with head and neck trauma. Cembranos et al. detected a piece of shrapnel that remained in the parotid gland for 63 years in their cases. In this case, the FB reaction suggested a tumoral lesion at the time of admission [3]. In the differential diagnosis of parotid FBs, sialadenitis, sialolithiasis, mucous plugs, and tumoral lesions should be considered [9].

Treatment of penetrating parotid FBs is early surgical exploration [3]. Removal of the FB becomes complex due to the course of the facial nerve in the parotid gland. Following the parotidectomy incision, it may be necessary to identify the facial nerve and reach the FB by careful dissection. A facial nerve stimulator can also be used during these procedures. There are different views about the approach to firearm injury of the parotid gland. Tissue damage is limited in low-energy firearm injuries, and there are those who accept that firearm parts such as bullets are sterile and can be monitored without removing the FB, as long as there are no symptoms [10]. However, it should not be forgotten that removing the FB from the parotid gland can prevent possible complications [3,10]. In our case, although the torpedo-shaped firecracker that caused the injury can be considered in the firearm class, it involves low energy and the scattering of plastic parts instead of metal parts may have caused the superficial penetration of the FB.

## Conclusions

Parotid gland FBs are extremely rare. They could be secondary to penetrating injury or could reach the parotid gland by retrograde migration. It should not be forgotten that there may be an FB lodged under the skin, especially in injuries related to explosive substances. Patients with recurrent symptoms in the same region after initial treatment should be investigated for FBs. This case report is the first in the literature in terms of the type of FB that causes injury to the parotid gland.
